# Supplementary material for: Genome-wide association study of dry eye disease reveals shared heritability with systemic comorbidities
Source: medRxiv. 2025 Mar 20:2025.03.18.25324218. Preprint. [Version 1] doi: 10.1101/2025.03.18.25324218 (PMC11957100; doi:10.1101/2025.03.18.25324218)
Supplement: Supplement 1 — Supplementary Information. VA Million Veteran Program: Core Acknowledgements Figure S1. Comparison of polygenic score associations across ancestries. Disease-related polygenic risk scores from the PGS Catalog were calculated in MVP and associated with dry eye disease case-control status in each ancestry. For comparison purposes, scores for the same phenotype were de-duplicated by semantic mapping to EFO terms, selecting the score for a harmonized EFO phenotype that was most significant in EUR. Correlation coefficients were calculated over all de-duplicated scores and over those with FDR < 0.05 significance in both ancestries. a) EUR (87,444 cases and 258,228 controls) versus AFR (30,734 cases and 58,335 controls) PGS z-scores. b) Comparison of EUR and AMR (12,940 cases and 31,864 controls) PGS z-scores. Figure S2. GWAS Q-Q plots. Genomic control inflation values (λ) are provided for each GWAS. a) EUR (87,444 cases and 258,228 controls). b) AFR (30,734 cases and 58,335 controls). c) AMR (12,940 cases and 31,864 controls). d) EAS (1,519 cases and 3,774 controls). e) Multi-ancestry meta-analysis (132,637 cases and 352,201 controls). Figure S4. Comparison of effect sizes at GWAS loci between MVP and the GERA replication cohorts. a) MVP multi-ancestry meta-analysis (132,637 cases and 352,201 controls) versus the GERA broad phenotype (16,025 cases and 54,818 controls). b) MVP multi-ancestry meta-analysis versus the stricter GERA narrow phenotype (3,317 cases and 54,516 controls). Log odds ratios with 95% confidence intervals are shown. One GWAS locus (rs191549504, at SYNGAP1) was not genotyped in GERA. The MLLT10 locus (rs12779865) is labeled. Figure S5. Comparison of effect size and allele frequency for GWAS loci. Scatter plot of odds ratio versus risk allele frequency for risk loci in the multi-ancestry meta-analysis (132,637 cases and 352,201 controls). The blue line denotes 80% power to achieve genome-wide significance. Independent genome-wide significant loci are hig [file media-1.pdf]

SUPPLEMENTARY INFORMATION FOR:

Genome-wide association study of dry eye disease reveals shared heritability with systemic comorbidities

Bryan R. Gorman[1,*,‡], Jaxon J. Huang[2,3,*], Peter B. Barr[4,5,6,7], Christopher W. Halladay[8], Cari L. Nealon[9,10], Chris Chatzinakos[5,6,4], Michael Francis[1], Chen Jiang[11], Million Veteran Program, Paul B. Greenberg[12,13], Wen-Chih Wu[14], Saiju Pyarajan[1], Hélène Choquet[11,15], Tim B. Bigdeli[4,5,6,7], Sudha K. Iyengar[16,17,18,†], Neal S. Peachey[18,19,20,†], Anat Galor[2,3,†,‡]

[1]Center for Data and Computational Sciences (C-DACS), VA Boston Healthcare System, 150 S Huntington Avenue, Boston, MA, 02130, USA, [2]Surgical and Research Services, Miami Veterans Administration Medical Center, 1201 NW 16th Street, Miami, FL, 33125, USA, [3]Bascom Palmer Eye Institute, University of Miami, 900 NW 17th Street, Miami, FL, 33136, USA, [4]Research Service, VA New York Harbor Healthcare System, Brooklyn, NY, 11209, USA, [5]Department of Psychiatry and Behavioral Sciences, SUNY Downstate Health Sciences University, Brooklyn, NY, 11203, USA, [6]Institute for Genomics in Health, SUNY Downstate Health Sciences University, Brooklyn, NY, 11203, USA, [7]Epidemiology & Biostatistics, School of Public Health, SUNY Downstate Health Sciences University, Brooklyn, NY, 11203, USA, [8]Center of Innovation in Long Term Services and Supports, Providence VA Medical Center, 830 Chalkstone Avenue, Providence, RI, 02908, USA, [9]Eye Clinic, VA Northeast Ohio Healthcare System, 10701 East Boulevard, Cleveland, OH, 44106, USA, [10]Ophthalmology & Visual Sciences, Case Western Reserve University School of Medicine, Cleveland, OH, 44106, USA, [11]Division of Research, Kaiser Permanente Northern California (KPNC), 4480 Hacienda Drive, Pleasanton, CA, 94588, USA, [12]Ophthalmology Section, Providence VA Medical Center, 830 Chalkstone Avenue, Providence, RI, 02909, USA, [13]Division of Ophthalmology, Alpert Medical School, Brown University, Providence, RI, 02903, USA, [14]Cardiology Section, Medical Service, Providence VA Medical Center, 830 Chalkstone Avenue, Providence, RI, 02908, USA, [15]Department of Health Systems Science, Kaiser Permanente Bernard J. Tyson School of Medicine, Pasadena, CA, 91101, USA, [16]Cleveland Institute for Computational Biology, Case Western Reserve University, Cleveland, OH, 44106, USA, [17]Department of Population and Quantitative Health Sciences, Case Western Reserve University School of Medicine, Cleveland, OH, 44106, USA, [18]Research Service, VA Northeast Ohio Healthcare System, 10701 East Boulevard, Cleveland, OH, 44106, USA, [19]Cole Eye Institute, Cleveland Clinic Foundation, 9500 Euclid Avenue, Cleveland, OH, 44195, USA, [20]Department of Ophthalmology, Cleveland Clinic Lerner College of Medicine of Case Western Reserve University, 9500 Euclid Avenue, Cleveland, OH, 44195, USA, *These authors contributed equally: B.R.G., J.J.H., †These authors jointly supervised this work: S.K.I., N.S.P., A.G., ‡Corresponding authors: gorman@alum.mit.edu; AGalor@med.miami.edu

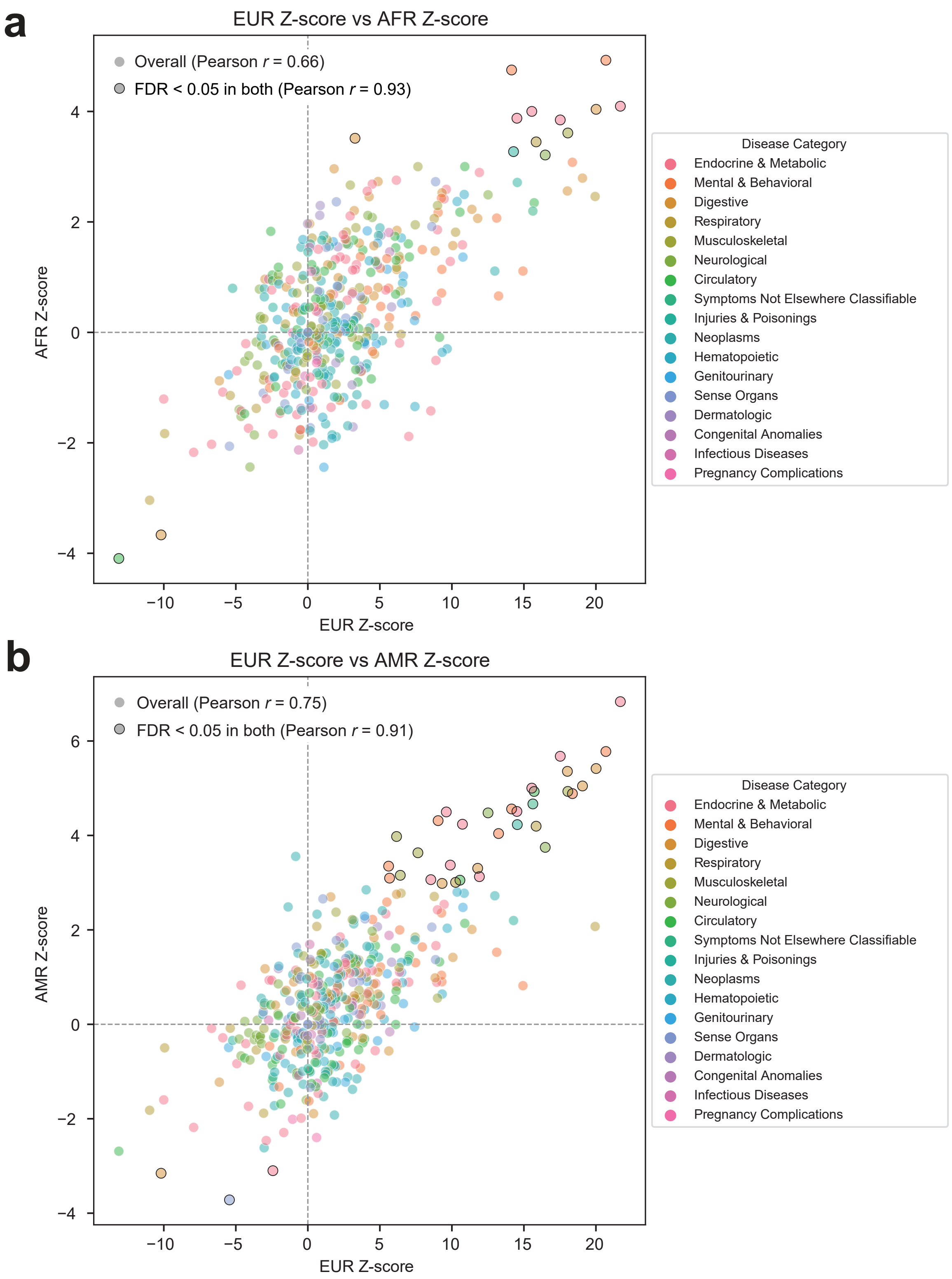

Figure S1. Comparison of polygenic score associations across ancestries. Disease-related polygenic risk scores from the PGS Catalog were calculated in MVP and associated with dry eye disease case-control status in each ancestry. For comparison purposes, scores for the same phenotype were de-duplicated by semantic mapping to EFO terms, selecting the score for a harmonized EFO phenotype that was most significant in EUR. Correlation coefficients were calculated over all de-duplicated scores and over those with FDR < 0.05 significance in both ancestries. **a)** EUR (87,444 cases and 258,228 controls) versus AFR (30,734 cases and 58,335 controls) PGS z-scores. **b)** Comparison of EUR and AMR (12,940 cases and 31,864 controls) PGS z-scores.

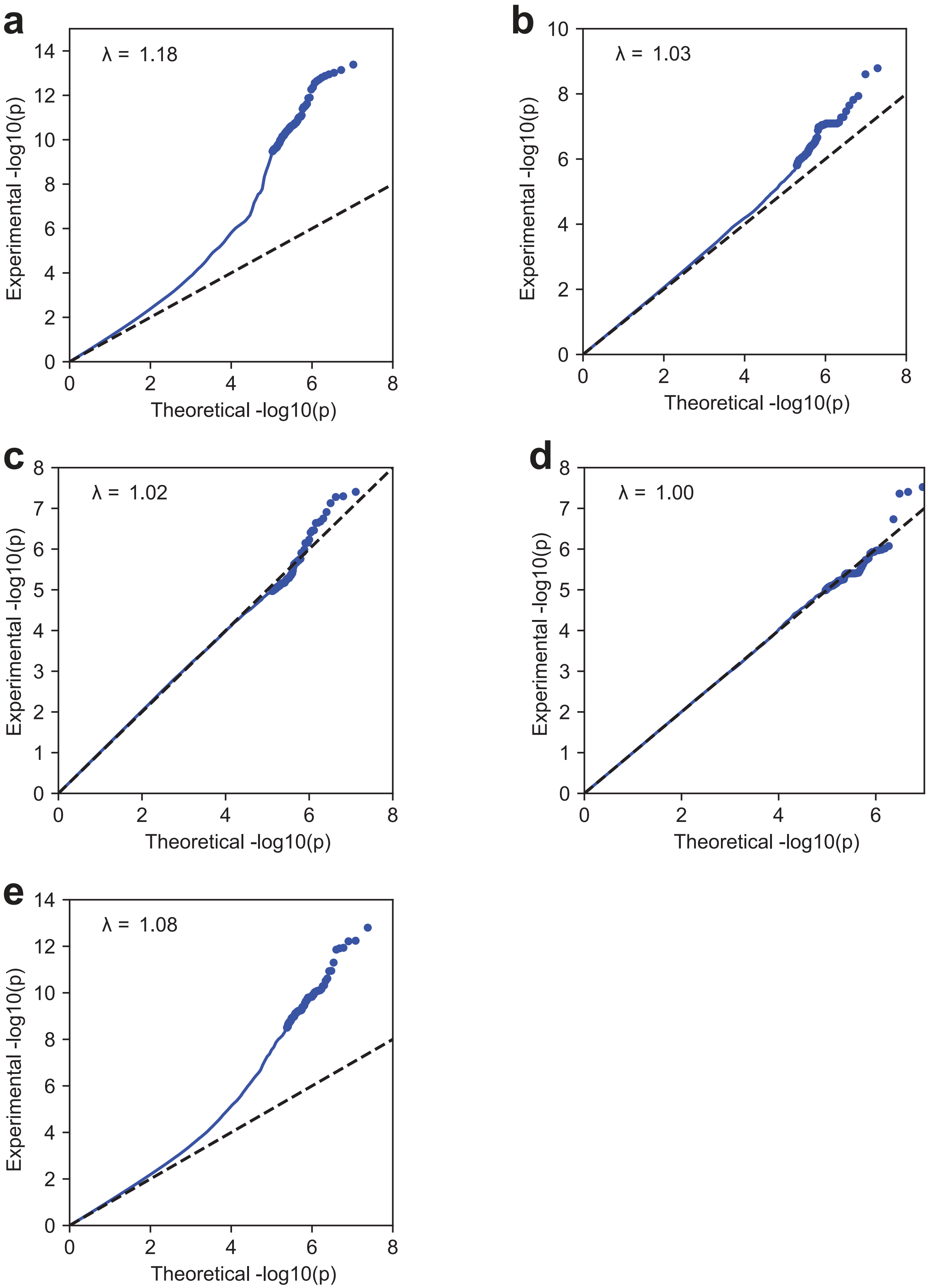

Figure S2. GWAS Q-Q plots. Genomic control inflation values (λ) are provided for each GWAS. **a**) EUR (87,444 cases and 258,228 controls). **b**) AFR (30,734 cases and 58,335 controls). **c**) AMR (12,940 cases and 31,864 controls). **d**) EAS (1,519 cases and 3,774 controls). **e**) Multi-ancestry meta-analysis (132,637 cases and 352,201 controls).

Figure S3. Regional association plots of significant loci from the multi-ancestry meta-analysis. 1000 Genomes EUR samples were used for the LD reference.

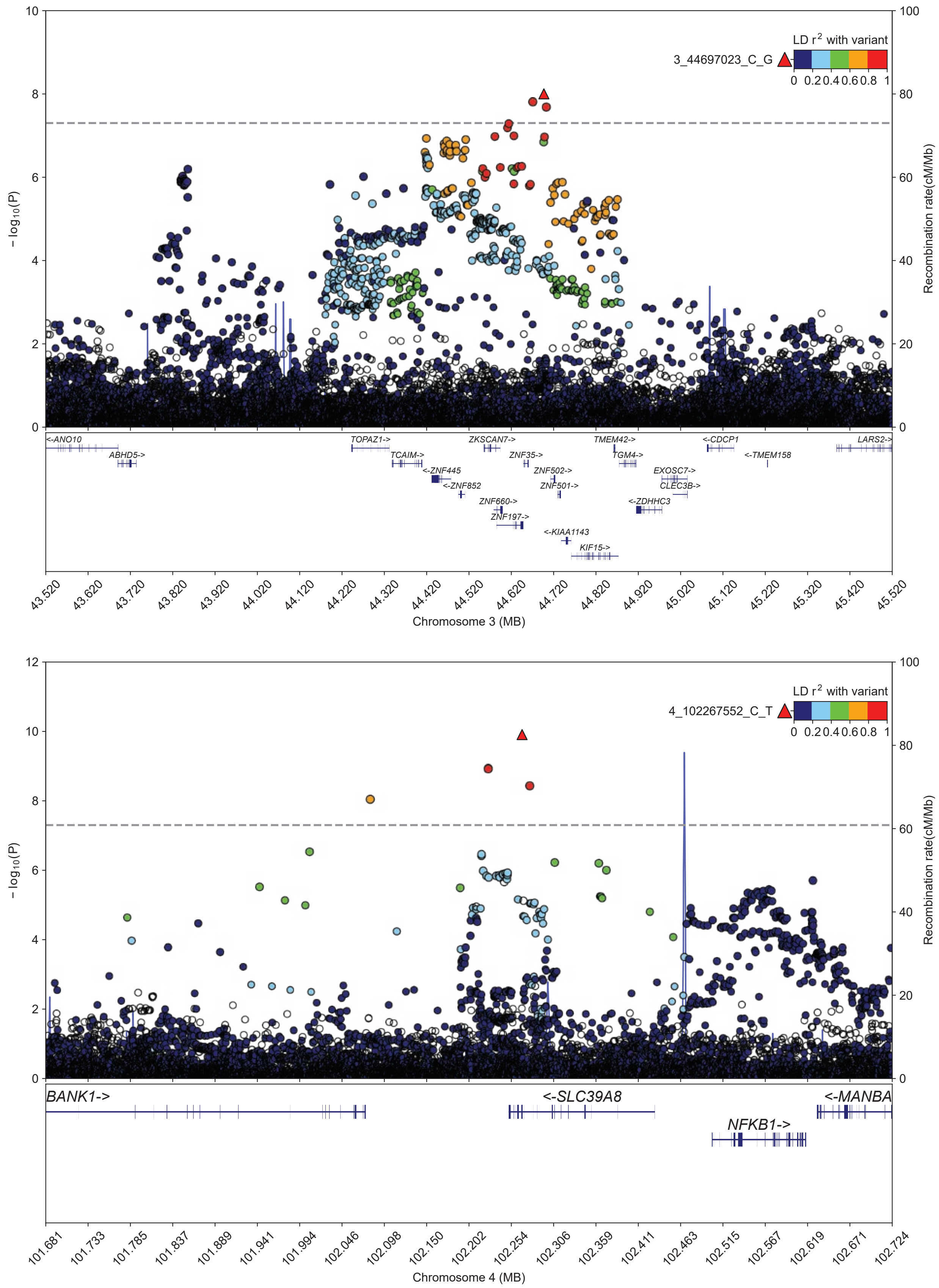

S3

c

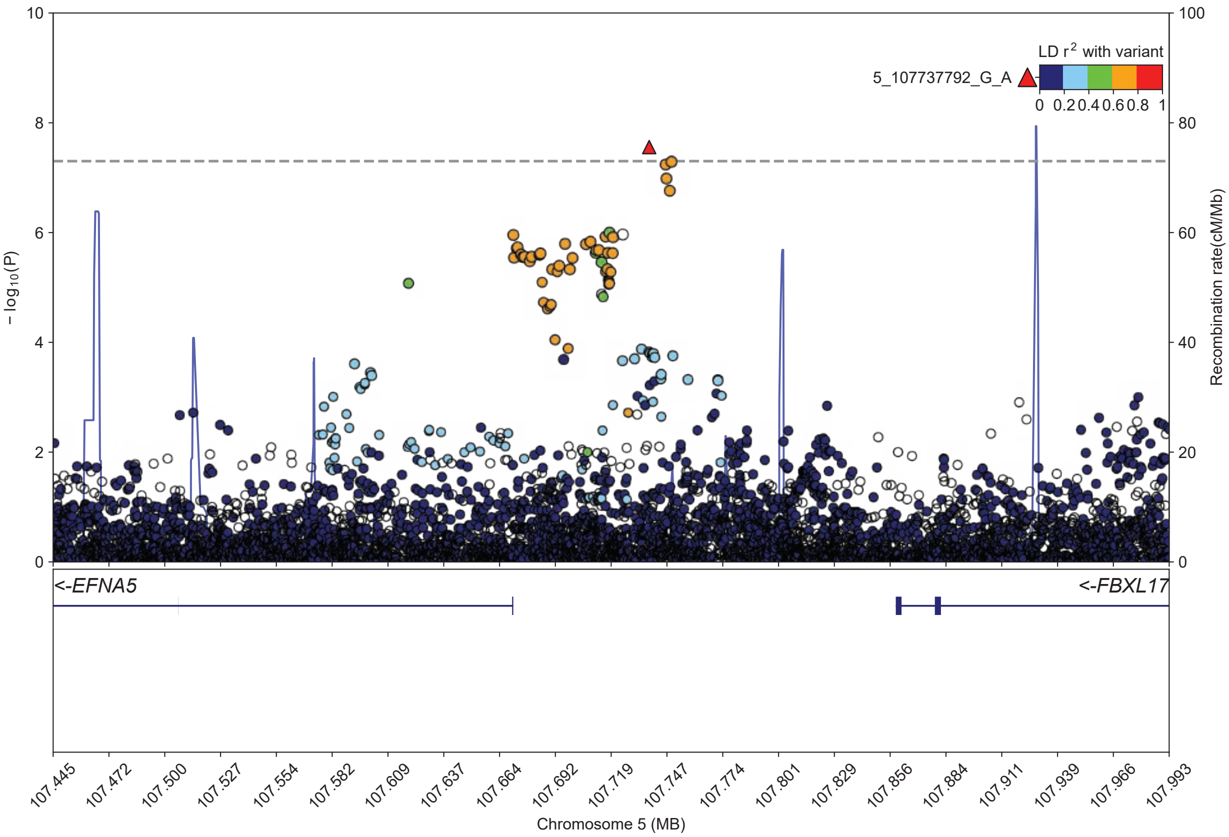

LD r² with variant
5_107737792_G_A
0 0.2 0.4 0.6 0.8 1
− log₁₀(P)
Recombination rate(cM/Mb)
<-EFNA5
<-FBXL17
107.445 107.472 107.500 107.527 107.554 107.582 107.609 107.637 107.664 107.692 107.719 107.747 107.774 107.801 107.829 107.856 107.884 107.911 107.939 107.966 107.993
Chromosome 5 (MB)

d

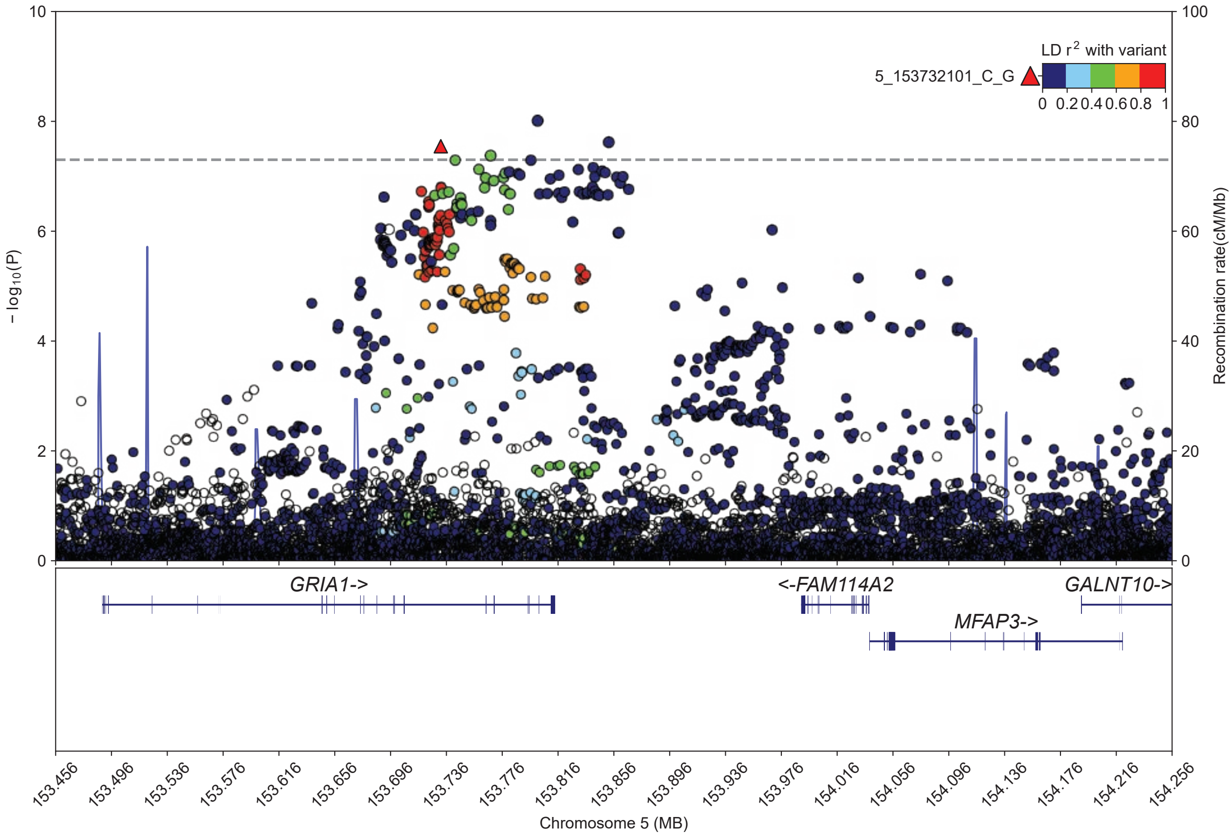

LD r² with variant
5_153732101_C_G
0 0.2 0.4 0.6 0.8 1
− log₁₀(P)
Recombination rate(cM/Mb)
GRIA1->
<-FAM114A2
GALNT10->
MFAP3->
153.456 153.496 153.536 153.576 153.616 153.656 153.696 153.736 153.776 153.816 153.856 153.896 153.936 153.976 154.016 154.056 154.096 154.136 154.176 154.216 154.256
Chromosome 5 (MB)

S3

e

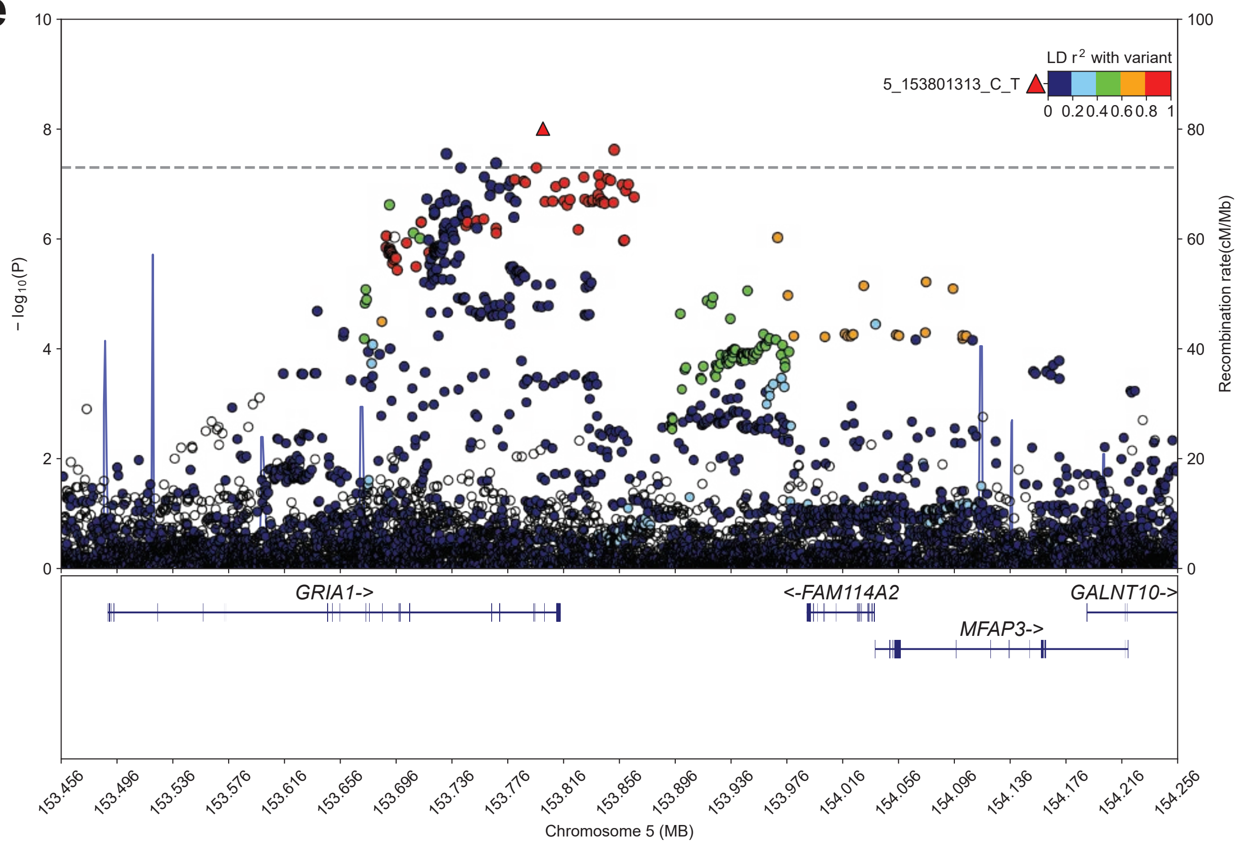
LD r2 with variant
5_153801313_C_T
0 0.2 0.4 0.6 0.8 1
− log10(P)
Recombination rate(cM/Mb)
GRIA1->
<-FAM114A2
GALNT10->
MFAP3->
Chromosome 5 (MB)

f

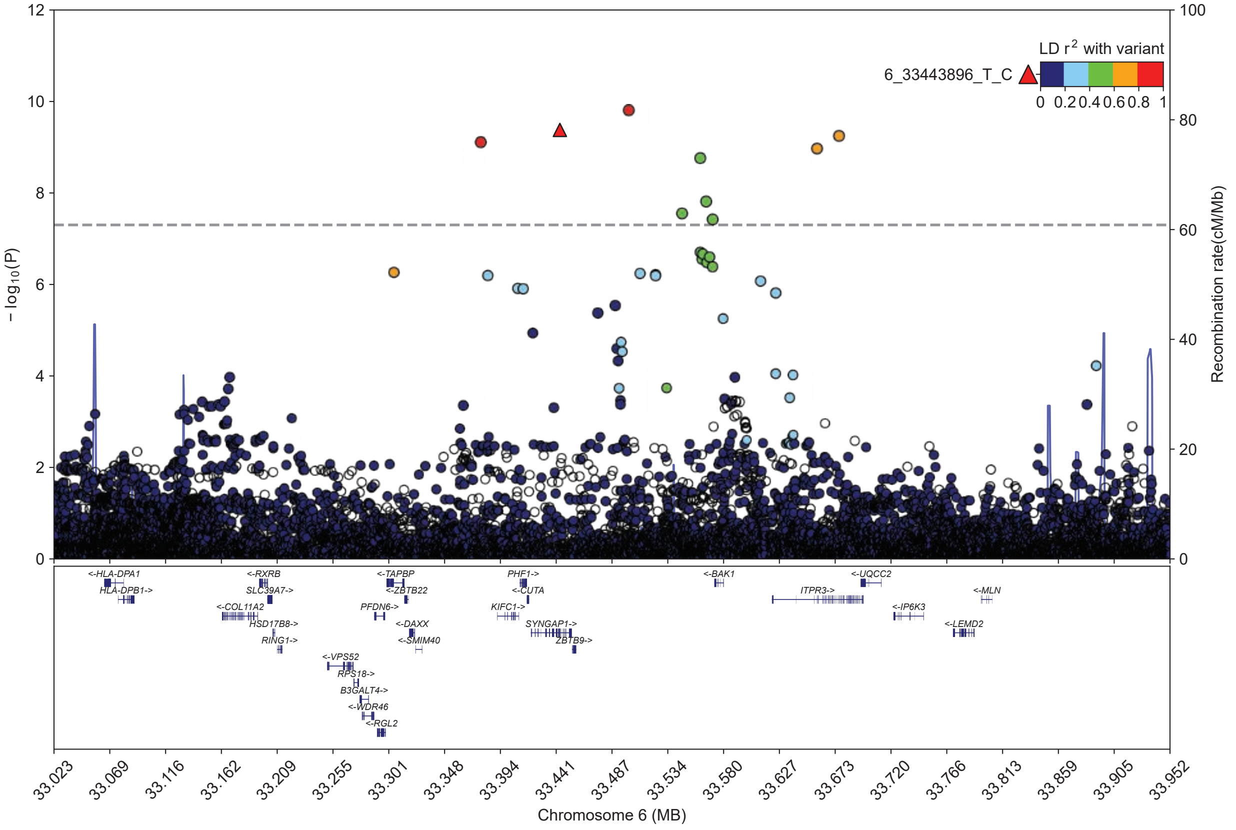
LD r2 with variant
6_33443896_T_C
0 0.2 0.4 0.6 0.8 1
− log10(P)
Recombination rate(cM/Mb)
Chromosome 6 (MB)

S3

g

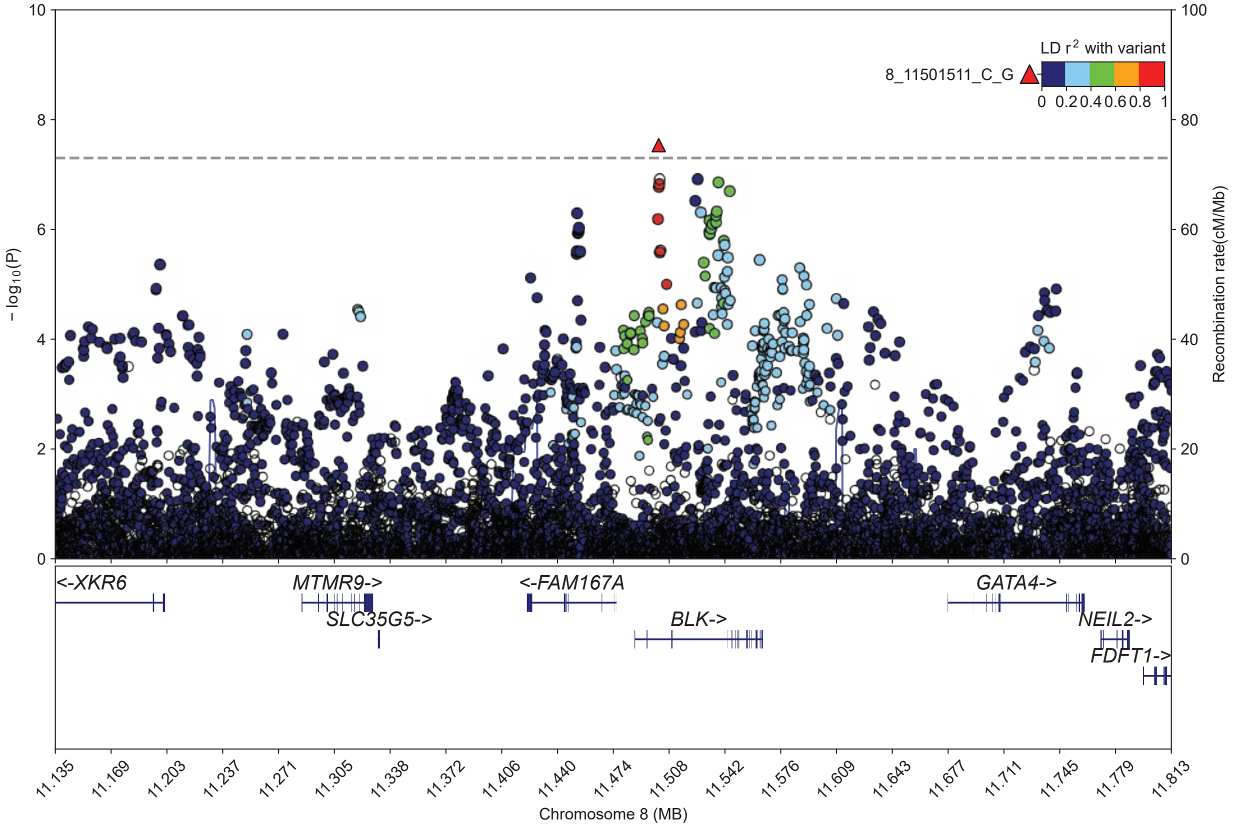
LD r2 with variant
8_11501511_C_G
0 0.2 0.4 0.6 0.8 1
− log10(P)
Recombination rate(cM/Mb)
<-XKR6
MTMR9->
SLC35G5->
<-FAM167A
BLK->
GATA4->
NEIL2->
FDFT1->
Chromosome 8 (MB)

h

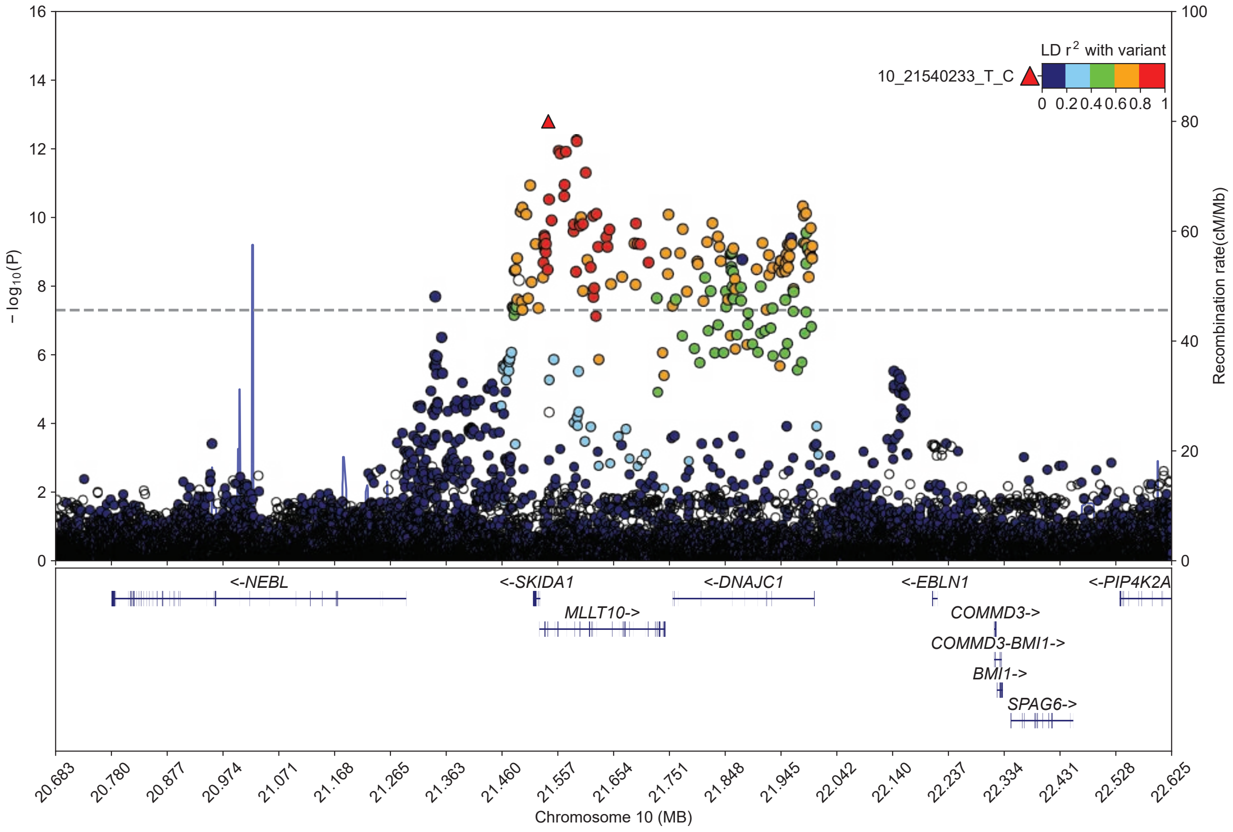
LD r2 with variant
10_21540233_T_C
0 0.2 0.4 0.6 0.8 1
− log10(P)
Recombination rate(cM/Mb)
<-NEBL
<-SKIDA1
MLLT10->
<-DNAJC1
<-EBLN1
COMMD3->
COMMD3-BMI1->
BMI1->
SPAG6->
<-PIP4K2A
Chromosome 10 (MB)

S3

i

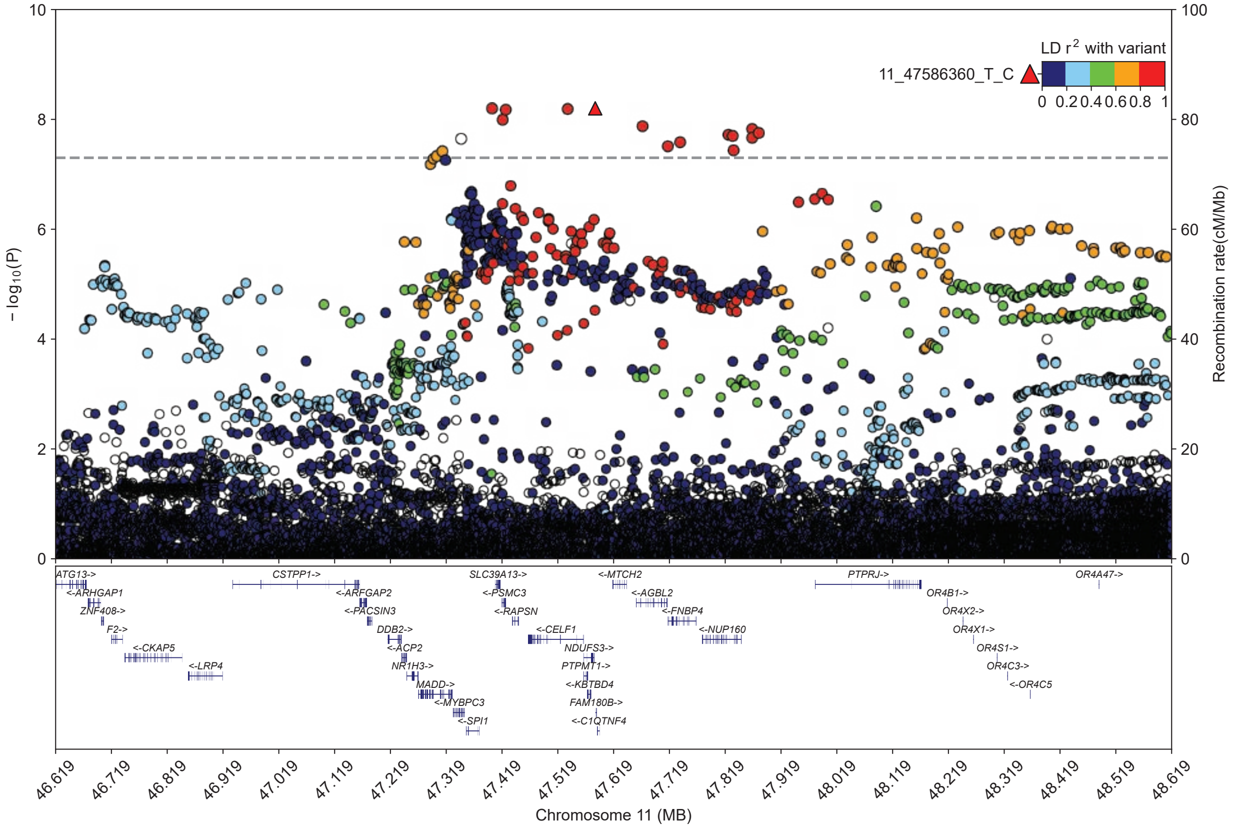

LD r2 with variant
11_47586360_T_C
0 0.2 0.4 0.6 0.8 1
− log10(P)
Recombination rate(cM/Mb)
ATG13->
<-ARHGAP1
ZNF408->
F2->
<-CKAP5
<-LRP4
CSTPP1->
<-ARFGAP2
<-PACSIN3
DDB2->
<-ACP2
NR1H3->
MADD->
<-MYBPC3
<-SPI1
SLC39A13->
<-PSMC3
<-RAPSN
<-CELF1
NDUFS3->
PTPMT1->
<-KBTBD4
FAM180B->
<-C1QTNF4
<-MTCH2
<-AGBL2
<-FNBP4
<-NUP160
PTPRJ->
OR4B1->
OR4X2->
OR4X1->
OR4S1->
OR4C3->
<-OR4C5
OR4A47->
Chromosome 11 (MB)

j

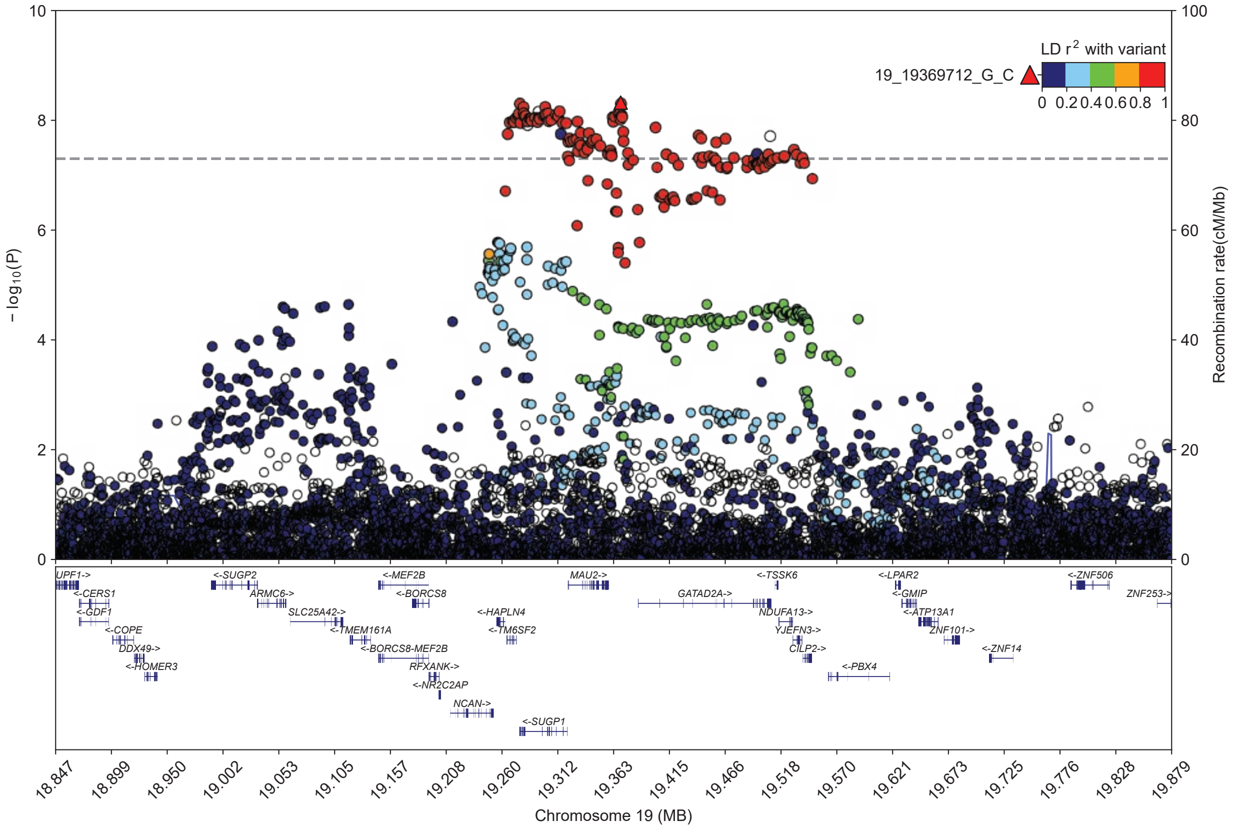

LD r2 with variant
19_19369712_G_C
0 0.2 0.4 0.6 0.8 1
− log10(P)
Recombination rate(cM/Mb)
UPF1->
<-CERS1
<-GDF1
<-COPE
DDX49->
<-HOMER3
<-SUGP2
ARMC6->
SLC25A42->
<-TMEM161A
<-BORCS8-MEF2B
<-MEF2B
<-BORCS8
RFXANK->
<-NR2C2AP
NCAN->
<-HAPLN4
<-TM6SF2
<-SUGP1
MAU2->
GATAD2A->
<-TSSK6
NDUFA13->
YJEFN3->
CILP2->
<-PBX4
<-LPAR2
<-GMIP
<-ATP13A1
ZNF101->
<-ZNF14
<-ZNF506
ZNF253->
Chromosome 19 (MB)

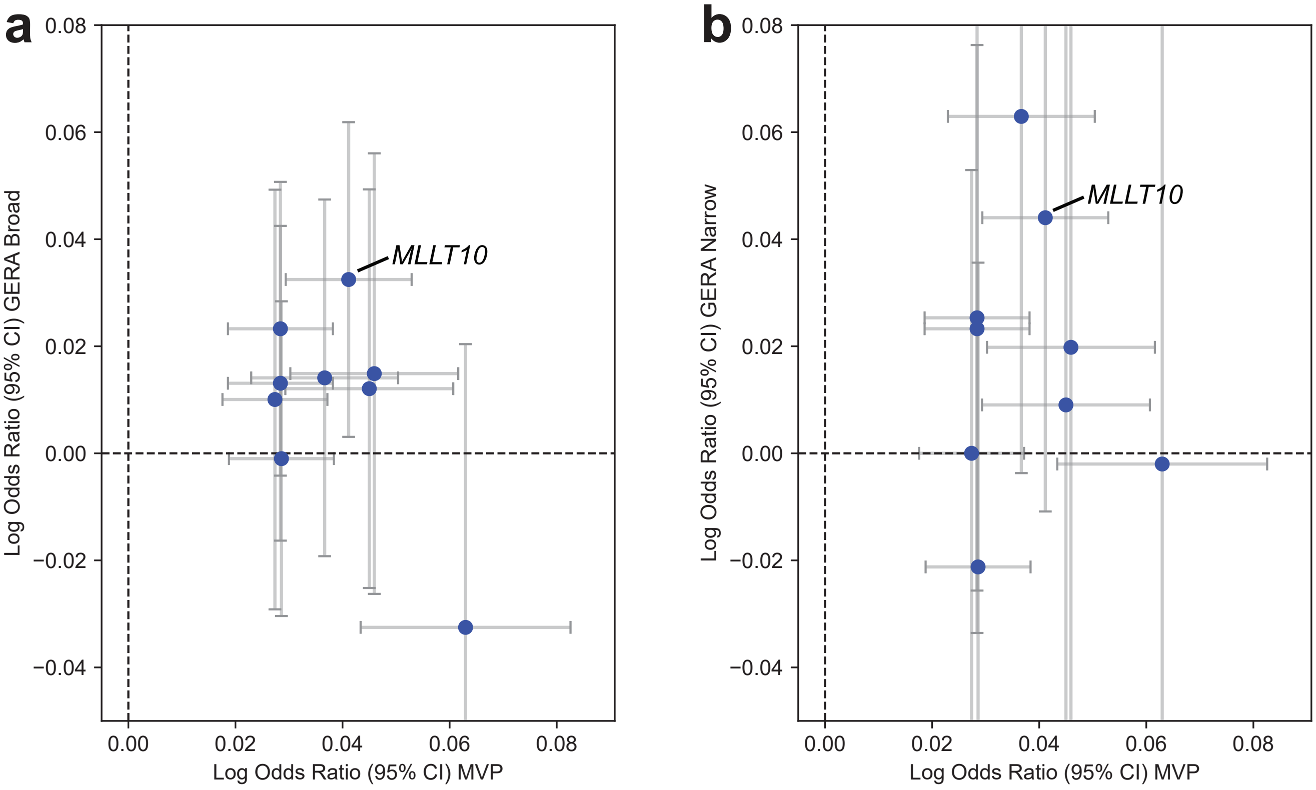

Figure S4. Comparison of effect sizes at GWAS loci between MVP and the GERA replication cohorts. a) MVP multi-ancestry meta-analysis (132,637 cases and 352,201 controls) versus the GERA *broad* phenotype (16,025 cases and 54,818 controls). **b)** MVP multi-ancestry meta-analysis versus the stricter GERA *narrow* phenotype (3,317 cases and 54,516 controls). Log odds ratios with 95% confidence intervals are shown. One GWAS locus (rs191549504, at *SYNGAP1*) was not genotyped in GERA. The *MLLT10* locus (rs12779865) is labeled.

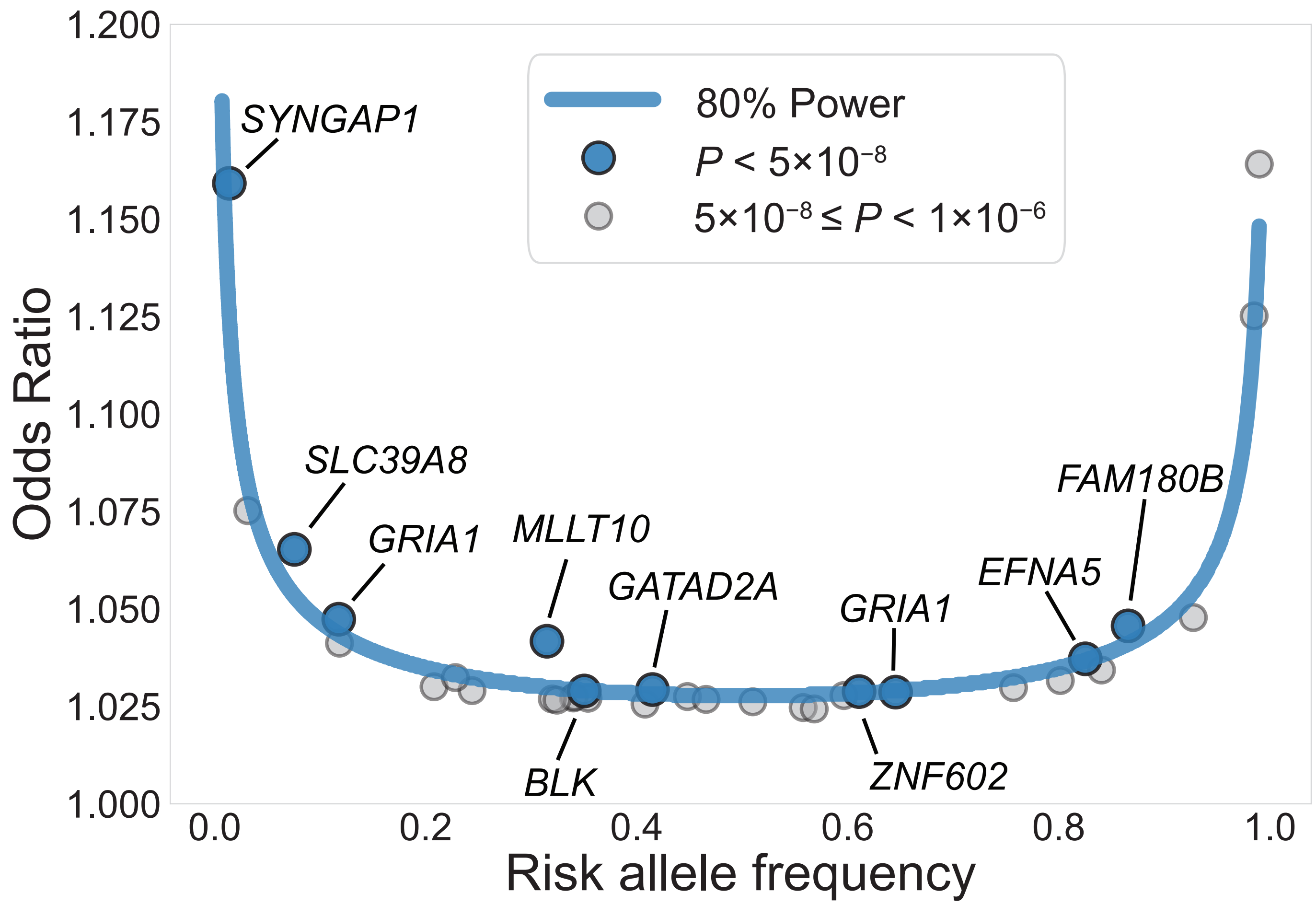

Figure S5. Comparison of effect size and allele frequency for GWAS loci. Scatter plot of odds ratio versus risk allele frequency for risk loci in the multi-ancestry meta-analysis (132,637 cases and 352,201 controls). The blue line denotes 80% power to achieve genome-wide significance. Independent genome-wide significant loci are highlighted in blue and labeled by nearest gene. Additional independent loci reaching a suggestive significance threshold ($P < 1{\times}10^{-6}$) are shown in gray.

VA Million Veteran Program:
Core Acknowledgements for Publications
May 2024

MVP Program Office

- Sumitra Muralidhar, Ph.D., Program Director
  US Department of Veterans Affairs, 810 Vermont Avenue NW, Washington, DC 20420
- Jennifer Moser, Ph.D., Associate Director, Scientific Programs
  US Department of Veterans Affairs, 810 Vermont Avenue NW, Washington, DC 20420
- Jennifer E. Deen, B.S., Associate Director, Cohort & Public Relations
  US Department of Veterans Affairs, 810 Vermont Avenue NW, Washington, DC 20420

MVP Executive Committee

- Co-Chair: Philip S. Tsao, Ph.D.
  VA Palo Alto Health Care System, 3801 Miranda Avenue, Palo Alto, CA 94304
- Co-Chair: Sumitra Muralidhar, Ph.D.
  US Department of Veterans Affairs, 810 Vermont Avenue NW, Washington, DC 20420
- J. Michael Gaziano, M.D., M.P.H.
  VA Boston Healthcare System, 150 S. Huntington Avenue, Boston, MA 02130
- Elizabeth Hauser, Ph.D.
  Durham VA Medical Center, 508 Fulton Street, Durham, NC 27705
- Amy Kilbourne, Ph.D., M.P.H.
  VA HSR&D, 2215 Fuller Road, Ann Arbor, MI 48105
- Michael Matheny, M.D., M.S., M.P.H.
  VA Tennessee Valley Healthcare System, 1310 24th Ave. South, Nashville, TN 37212
- Dave Oslin, M.D.
  Philadelphia VA Medical Center, 3900 Woodland Avenue, Philadelphia, PA 19104
- Deepak Voora, MD
  Durham VA Medical Center, 508 Fulton Street, Durham, NC 27705

MVP Co-Principal Investigators

- J. Michael Gaziano, M.D., M.P.H.
  VA Boston Healthcare System, 150 S. Huntington Avenue, Boston, MA 02130
- Philip S. Tsao, Ph.D.
  VA Palo Alto Health Care System, 3801 Miranda Avenue, Palo Alto, CA 94304

MVP Core Operations

- Jessica V. Brewer, M.P.H., Director, MVP Cohort Operations
  VA Boston Healthcare System, 150 S. Huntington Avenue, Boston, MA 02130
- Mary T. Brophy M.D., M.P.H., Director, VA Central Biorepository
  VA Boston Healthcare System, 150 S. Huntington Avenue, Boston, MA 02130
- Kelly Cho, M.P.H, Ph.D., Director, MVP Phenomics

VA Boston Healthcare System, 150 S. Huntington Avenue, Boston, MA 02130
- Lori Churby, B.S., Director, MVP Regulatory Affairs
  VA Palo Alto Health Care System, 3801 Miranda Avenue, Palo Alto, CA 94304
- Scott L. DuVall, Ph.D., Director, VA Informatics and Computing Infrastructure (VINCI)
  VA Salt Lake City Health Care System, 500 Foothill Drive, Salt Lake City, UT 84148
- Saiju Pyarajan Ph.D., Director, Data and Computational Sciences
  VA Boston Healthcare System, 150 S. Huntington Avenue, Boston, MA 02130
- Robert Ringer, Pharm.D., Director, VA Albuquerque Central Biorepository
  New Mexico VA Health Care System, 1501 San Pedro Drive SE, Albuquerque, NM 87108
- Luis E. Selva, Ph.D., Director, MVP Biorepository Coordination
  VA Boston Healthcare System, 150 S. Huntington Avenue, Boston, MA 02130
- Shahpoor (Alex) Shayan, M.S., Director, MVP PRE Informatics
  VA Boston Healthcare System, 150 S. Huntington Avenue, Boston, MA 02130
- Brady Stephens, M.S., Principal Investigator, MVP Information Center
  Canandaigua VA Medical Center, 400 Fort Hill Avenue, Canandaigua, NY 14424
- Stacey B. Whitbourne, Ph.D., Director, MVP Cohort Development and Management
  VA Boston Healthcare System, 150 S. Huntington Avenue, Boston, MA 02130

MVP Publications and Presentations Committee

- Co-Chair: Themistocles L. Assimes, M.D., Ph. D
  VA Palo Alto Health Care System, 3801 Miranda Avenue, Palo Alto, CA 94304
- Co-Chair: Adriana Hung, M.D.; M.P.H
  VA Tennessee Valley Healthcare System, 1310 24th Ave. South, Nashville, TN 37212
- Co-Chair: Henry Kranzler, M.D.
  Philadelphia VA Medical Center, 3900 Woodland Avenue, Philadelphia, PA 19104
